# Unidentified but Not Unknown: Evaluating the Phonetic Naming System in a UK Trauma Centre

**DOI:** 10.7759/cureus.99127

**Published:** 2025-12-13

**Authors:** Uday Mahajan, Ashit Mitra, Ria Gupta, Edward Spurrier, Vibhore Gupta, Krishnakumar Subbaraman

**Affiliations:** 1 Trauma and Orthopaedics, Queen Elizabeth Hospital Birmingham, Birmingham, GBR; 2 Emergency Medicine, Queen Elizabeth Hospital Birmingham, Birmingham, GBR; 3 Emergency Medicine, University of Birmingham, Birmingham, GBR; 4 Emergency, Queen Elizabeth Hospital Birmingham, Birmingham, GBR

**Keywords:** alias naming, major trauma, patient safety, staff perceptions, unidentified patients

## Abstract

Background

Unidentified or “unknown” patients present significant safety challenges in emergency and trauma care. Structured alias systems aim to reduce misidentification, but practical issues in their use are not well understood. This review evaluated local naming practices for unidentified patients and explored staff experiences with the phonetic alias system at a UK tertiary trauma centre.

Methods

A mixed-methods review combined policy analysis, a three-month audit of trauma admissions, and an anonymous staff survey (n = 25). The survey captured professional role, experience, perceived safety, training adequacy, and operational challenges. Quantitative data were analysed descriptively, and free-text comments underwent thematic analysis.

Results

Most unidentified patients were young male trauma cases requiring airway support. The NATO phonetic naming system was consistently applied and reduced duplication, but documentation of alias assignment and record merging was inconsistent. Staff generally perceived the system as safe (72% rated “good/excellent”), though only 40% felt adequately trained. Free-text responses highlighted confusion between similar-sounding aliases, insufficient training, record-merging errors, and the need for clearer protocols and improved electronic integration.

Conclusion

Structured phonetic naming supports patient safety, but remains vulnerable to human and process variability. Standardised guidance, embedded training, and better digital integration could strengthen reliability, and ensure unidentified-patient management remains safe, efficient, and respectful.

## Introduction

Accurate patient identification is fundamental to safe clinical care, particularly in emergency and trauma settings, where patients often arrive unconscious, intubated, or without documentation [1,2]. In such circumstances, temporary aliases are required to enable rapid registration and treatment. Traditional placeholder systems, such as “John Doe” or “Unknown Patient,” have been used for decades, but are prone to duplication, misidentification, and communication errors [3,4]. These risks are compounded in high-volume trauma centres, where multiple unidentified patients may be managed simultaneously [5,6].

To mitigate these risks, structured alias systems have been developed to ensure that each patient receives a unique and recognisable identifier. Robinson et al. (1985) proposed a numbered alias framework using pre-assigned hospital identifiers, while Brooks et al. (1999) introduced a NATO phonetic alphabet system, combining a phonetic name with sex and admission date, to enhance clarity during verbal and written communication [6,7]. More recent studies, such as Rogers et al. (2024), have described the challenges of embedding alias systems into electronic health records, where data duplication and merging failures can still lead to safety incidents, such as transfusion or medication errors [8]. Collectively, these reports illustrate the ongoing challenge of designing systems that are simultaneously safe, efficient, and compatible with modern digital workflows.

In the United Kingdom, the importance of safe temporary identification has been formally recognised in the NHS Improvement Patient Safety Alert (2018), which outlined standards for randomised phonetic names, locally unique codes, and estimated dates of birth [9]. While this alert established a national framework for safer alias generation, there is little published evidence describing how these systems perform in real-world practice [10]. In particular, few studies have examined how consistently such policies are applied in hospital trauma settings, how effectively aliases are reconciled once a patient’s identity is confirmed, and how frontline staff perceive the usability and safety of these systems.

This practice review aimed to evaluate the effectiveness and safety of the current phonetic alias system used for unidentified patients in a UK tertiary trauma centre. The objectives were to (1) review existing hospital policy, (2) audit real-world use of aliases and record reconciliation, and (3) capture staff perspectives on safety, training, and areas for improvement. Together, these findings provide an evidence base to inform local improvement, and contribute to the broader discussion on best practice for unidentified-patient management.

## Materials and methods

This study was conducted as a practice review and service evaluation of the system used for naming unidentified patients at a UK tertiary trauma centre. The objective was to describe current practice, assess safety and operational challenges, and capture staff perspectives, to inform local improvement. In line with institutional governance policy, no patient-identifiable data were collected, and formal ethical approval was not required.

The review comprised three complementary components. First, a policy review examined institutional procedures for registering unidentified patients and assessed their alignment with national and international guidance. Second, a three-month retrospective audit of emergency and trauma admissions (June-August 2025) identified all cases registered under temporary aliases. Data collected included the alias structure used, the number of concurrent unidentified patients, time to verified identification, and any reported issues, such as duplication, merging delays, or documentation gaps within the electronic health record.

Finally, an anonymous online staff survey was distributed to clinical and administrative staff working in the emergency and trauma departments. The survey consisted of 12 closed and two open-ended questions, addressing professional role, experience, frequency of unidentified-patient encounters, perceived safety, workflow efficiency, communication, and adequacy of training. Free-text items invited examples of challenges or suggestions for improvement. Quantitative data were analysed descriptively using Microsoft Excel (Microsoft® Corp., Redmond, WA, USA), and qualitative responses underwent inductive thematic analysis to identify recurring patterns.

Given the exploratory nature of the project and the small sample size, no inferential statistical tests were applied. Results are presented as counts and percentages, to illustrate proportions and trends rather than to infer statistical significance. Findings from the policy review, audit, and survey were triangulated and compared with published literature to situate local practice within the wider evidence base.

## Results

Audit of unidentified trauma patients

Between June and August 2025, a total of 20 patients were registered under temporary trauma aliases. The majority were male (n = 16, 80%), with a median age of 34 years (range 14-76). Most presented following polytrauma, head injury, or penetrating trauma, and in all but two cases, the patients were not previously known to the hospital.

Aliases were generated using a structured NATO phonetic convention, typically combining a phonetic name with the hospital identifier “QEHB.” In two cases, an additional distinguishing element was appended. While the phonetic system ensured unique identifiers, documentation of the rationale for alias assignment was inconsistent. Common reasons for alias use included reduced consciousness, intubation on arrival, language barriers, or absence of identification.

In most cases (n = 15, 75%), trauma aliases were successfully merged with verified identities, though the timing of reconciliation varied and was poorly recorded. Identification was typically achieved through police or family confirmation, or when patients regained capacity. In one case, merging was not completed at all.

Complications were rare but clinically significant. One patient received an incorrect medication due to alias confusion. No transfusion errors were documented, though notes indicated that duplicate records and delayed test results occasionally occurred. At the time of data collection, four patients (20%) had died, 12 (60%) were discharged, three (15%) remained inpatients, and one (5%) had absconded. Table 1 provides a summary of survey details.

**Table 1:** Characteristics of unidentified trauma patients (n = 20)

Characteristic		n (%)
Gender	Male	16 (80)
	Female	4 (20)
Mechanism of injury	Polytrauma/road traffic collision (RTC)	8 (40)
	Head injury (isolated)	6 (30)
	Penetrating trauma	4 (20)
	Other	2 (10)
Reason for unidentified status	Reduced consciousness/intubated	9 (45)
	No ID/language barrier	5 (25)
	Found unaccompanied	4 (20)
	Other	2 (10)
Outcome at review	Discharged	12 (60)
	Inpatient	3 (15)
	Died	4 (20)
	Absconded	1 (5)

Staff survey findings

Free-text comments provided deeper insight into the quantitative findings. Staff widely regarded the phonetic alias system as logical and generally effective but noted challenges when several unidentified patients were admitted simultaneously. Similar-sounding aliases (e.g., “Alpha” and “Bravo”) were cited as a potential source of confusion in busy or noisy clinical environments. Respondents also highlighted insufficient induction or refresher training, particularly for rotating staff unfamiliar with the process, and reported that record-merging during identity reconciliation occasionally resulted in duplicated or lost results.

Suggestions for improvement centred on standardised training, clearer written protocols, and enhanced electronic integration to streamline the merging process and minimise manual error. Some participants proposed adding a secondary identifier, such as gender or approximate age, to make aliases more distinguishable. Overall, the comments reflected broad support for the phonetic naming system as a safety measure, tempered by recognition that its reliability depends on consistent application, staff awareness, and digital support. A summary of the staff survey results is presented in Table 2.

**Table 2:** Staff survey summary (n = 25)

Domain		Key findings, n (%)
Primary role	Physician	14 (56)
	Nurse	5 (20)
	Administrative/other	6 (24)
Experience in trauma care	>5 years	15 (60)
	≤5 years	10 (40)
Frequency of unidentified patient encounters	Daily or weekly	17 (68)
	Monthly or less	8 (32)
Ease of recognising aliases	Easy/very easy	16 (64)
	Difficult	5 (20)
	Unsure	4 (16)
Confusion or near-miss reported	Yes	4 (16)
	No	21 (84)
Patient safety rating - Good/excellent		18 (72)
Workflow efficiency - Good/excellent		17 (68)
Team communication - Good/excellent		18 (72)
Adequate training on alias use	Yes	10 (40)
	No	8 (32)
	Unsure	7 (28)

## Discussion

This practice review provides an integrated evaluation of unidentified-patient naming systems in a UK tertiary trauma centre. The findings show that the structured NATO phonetic convention is consistently applied, ensuring clear and unique identifiers, and reducing the duplication seen with unstructured systems such as “John Doe” [4,11,12]. However, documentation of alias assignment and merging to verified identities was inconsistent, and one clinically significant error related to misidentification occurred. These issues highlight that, although structured systems improve traceability, their effectiveness depends heavily on human reliability and procedural clarity.

The staff survey offered a complementary perspective, revealing strong overall confidence in the alias system’s purpose, but highlighting gaps in its operational consistency. Most respondents regarded the system as safe, yet some reported confusion when multiple phonetic names with similar sounds were active simultaneously. Limited formal training emerged as a key weakness, particularly for new or rotating staff. Problems with record merging were also common, echoing findings from international studies, where delayed or incomplete reconciliation has led to duplicated investigations and miscommunication [13]. Research by Rogers et al. (2024) and Janowak et al. (2019) similarly emphasises that unidentified patients remain at disproportionate risk of clerical and clinical error, despite the use of structured systems [8,14]. These findings complement the principles outlined in the NHS Improvement (2018) Patient Safety Alert, which advocated for randomised phonetic identifiers and inclusion of demographic markers; however, our results indicate that consistent implementation and staff familiarity remain challenging in practice.

These findings suggest that, while phonetic naming conventions enhance safety compared to generic placeholders, they are not immune to failure within complex, high-pressure trauma workflows [5,15,16]. Strengthening staff education and embedding alias protocols within electronic systems could reduce variability and improve the reliability of identity reconciliation. The introduction of simple supplementary identifiers, such as gender or age band, may further minimise confusion when several unidentified patients are admitted concurrently. From a broader perspective, development of a national standard for alias naming, integrated with electronic health records, could ensure cross-institutional consistency and improve continuity of care [11,12].

Beyond operational safety, the use of temporary aliases carries ethical implications related to dignity and identity. Although this review did not explore patient or family perspectives, previous studies suggest that placeholder names may be perceived as impersonal or distressing [8,14]. Rapid restoration of a verified identity should, therefore, be prioritised not only for safety but also for patient-centred care.

This study has limitations. It was conducted in a single centre with a modest survey sample, and alias records were captured retrospectively, meaning not all unidentified presentations may have been represented. The short survey format, designed for feasibility in a busy clinical environment, may not fully reflect the breadth of staff experiences. These factors limit generalisability; however, they provide an important baseline for future, larger-scale or multi-centre evaluations.

## Conclusions

The structured phonetic naming system provides a safe and practical framework for managing unidentified patients in trauma care, but its reliability depends on consistent implementation, clear documentation, and adequate staff training. Variability in practice, particularly around record merging and staff induction, remains a source of potential error. Strengthening education, embedding alias protocols within electronic systems, and developing a national standard for unidentified-patient naming would enhance safety, efficiency, and continuity of care. Future work should also explore patient and family perspectives to ensure that identification practices uphold both safety and dignity.
